# Cardioprotection by Preconditioning with Intralipid Is Sustained in a Model of Endothelial Dysfunction for Isolated-Perfused Hearts

**DOI:** 10.3390/ijms252010975

**Published:** 2024-10-12

**Authors:** Martin Stroethoff, Natalie Schneider, Lea Sung, Jan Wübbolt, André Heinen, Annika Raupach

**Affiliations:** 1 Department of Anesthesiology, Medical Faculty and University Hospital Düsseldorf, Heinrich-Heine University Düsseldorf, 40225 Düsseldorf, Germanyjan.wuebbolt@hhu.de (J.W.); 2 Institute for Cardiovascular Physiology, Medical Faculty and University Hospital Düsseldorf, Heinrich-Heine University Düsseldorf, 40225 Düsseldorf, Germany

**Keywords:** endothelial dysfunction, Intralipid, conditioning, cardioprotection, ischemia/reperfusion, Langendorff

## Abstract

Endothelial dysfunction (ED) is closely associated with most cardiovascular diseases. Experimental models are needed to analyze the potential impact of ED on cardioprotection in constant pressure Langendorff systems (CPLS). One cardioprotective strategy against ischemia/reperfusion injury (I/RI) is conditioning with the lipid emulsion Intralipid (IL). Whether ED modulates the cardioprotective effect of IL remains unknown. The aim of the study was to transfer a protocol using a constant flow Langendorff system for the induction of ED into a CPLS, without the loss of smooth muscle cell functionality, and to analyze the cardioprotective effect of IL against I/RI under ED. In isolated hearts of male Wistar rats, ED was induced by 10 min perfusion of a Krebs–Henseleit buffer containing 60 mM KCl (K+), and the vasodilatory response to the vasodilators histamine (endothelial-dependent) and sodium–nitroprusside (SNP, endothelial-independent) was measured. A CPLS was employed to determine cardioprotection of pre- or postconditioning with 1% IL against I/RI. The constant flow perfusion of K+ reduced endothelial response to histamine but not to SNP, indicating reduced vasodilatory functionality of endothelial cells but not smooth muscle cells. Preconditioning with IL reduced infarct size and improved cardiac function while postconditioning with IL had no effect. The induction of ED neither influenced infarct size nor affected the cardioprotective effect by preconditioning with IL. This protocol allows for studies of cardioprotective strategies under ED in CLPS. The protection by preconditioning with IL seems to be mediated independently of a functional endothelium.

## 1. Introduction

Cardiovascular diseases (CVD) are the leading cause of death worldwide and cause an enormous financial burden [1,2]. Therefore, further investigations of cardioprotective methods and interventions are needed to prevent the development and/or progression of CVD. In the last few decades, experimental studies using ischemic conditioning (IC) showed remarkable results in protecting against ischemia/reperfusion (I/R) injury, for example, nearly halving the infarct size after myocardial infarction in in vitro and in vivo models [3]. In these models, short cycles of I/R are performed before (pre-), during (peri-), or after (post-) severe and long ischemia, which do not cause defects by themselves due to their short duration. The cardioprotective effect of IC can also be mimicked by using drugs, called pharmacological conditioning. One such drug is the lipid emulsion Intralipid (IL). It has already been shown that postconditioning with IL is cardioprotective, resulting in improved heart function and reduced infarct size, using an in vivo rat model and in vitro mouse hearts [4,5]. Other groups’ findings supported these cardioprotective effects, for example in swine [6] or in remodeled rat hearts [7].

However, a successful transfer of cardioprotective conditioning from bench to bedside has not been established yet. The cause seems to be an inhibition of the cardioprotective effect by confounding factors like comedication, comorbidities, or associated pathological changes, for example in tissue function or signal transduction [8]. These confounders are numerous in patients with the need for cardioprotection because they are usually older, suffer from multiple diseases, and are treated with various medications. Most experimental studies, however, were performed in young, healthy animals without consideration of these confounding factors. Therefore, the development of model systems mimicking such patients is required to verify cardioprotective effects under more realistic conditions.

Most cardiovascular diseases and their typical comorbidities such as hypertension or diabetes mellitus are closely associated with endothelial dysfunction (ED) or are promoted by it [9]. ED is defined as the insufficient bioavailability of nitric oxide (NO) in the vasculature, leading to an imbalance in vascular tone with reduced endothelium-dependent relaxation [10,11]. Mainly, reduced NO production by the endothelial NO-synthase (eNOS) in the vessels is responsible for ED. A widely used ex vivo system to study cardioprotective mechanisms is the isolated perfused heart according to Langendorff. Two methods have been described for the induction of ED in this system: a bolus of triton (Triton X-100) and perfusion with buffers containing high amounts of potassium [12,13]. Triton acts via unspecific cell lysis, posing a high risk of destroying not only endothelial cells, which is a pivotal disadvantage compared to high potassium buffers. Electron microscopic pictures taken after 1 min of perfusion with 0.01% triton show damaged myocardium and coronary arteries with disrupted and detached endothelium, edema, and broken mitochondria [14]. However, high-potassium buffers apparently require shear stress for the induction of ED, demanding high perfusion flow [13,15,16]. To achieve this perfusion flow, a constant flow Langendorff system is commonly used. However, a constant pressure system has been highly recommended for studies investigating cardioprotective effects against I/R injury [17]. This is based on the potential interference of a constant flow system with the heart’s responses to ischemia. For example, during ischemic contraction, perfusion is completely interrupted even when using a constant pressure system and the pressure does not rise above the previously defined system pressure. In contrast, in constant flow mode, the perfusate would be further transported through the heart against the resistance of the vessels, which can lead to extremely high perfusion pressures and thus to significantly higher tissue damage. Therefore, a transfer of a published protocol for induction of ED via buffers containing high potassium amounts, established in a constant flow Langendorff system, into a constant pressure system is needed.

The aim of this present study was therefore (1) to transfer a protocol for the induction of ED without loss of functionality of smooth muscle cells into a constant pressure Langendorff system and (2) to prove this protocol by analyzing the cardioprotective effect of IL against I/R injury under ED.

## 2. Results

### 2.1. Protocol for Induction of Endothelial Dysfunction

**Figure 1:**
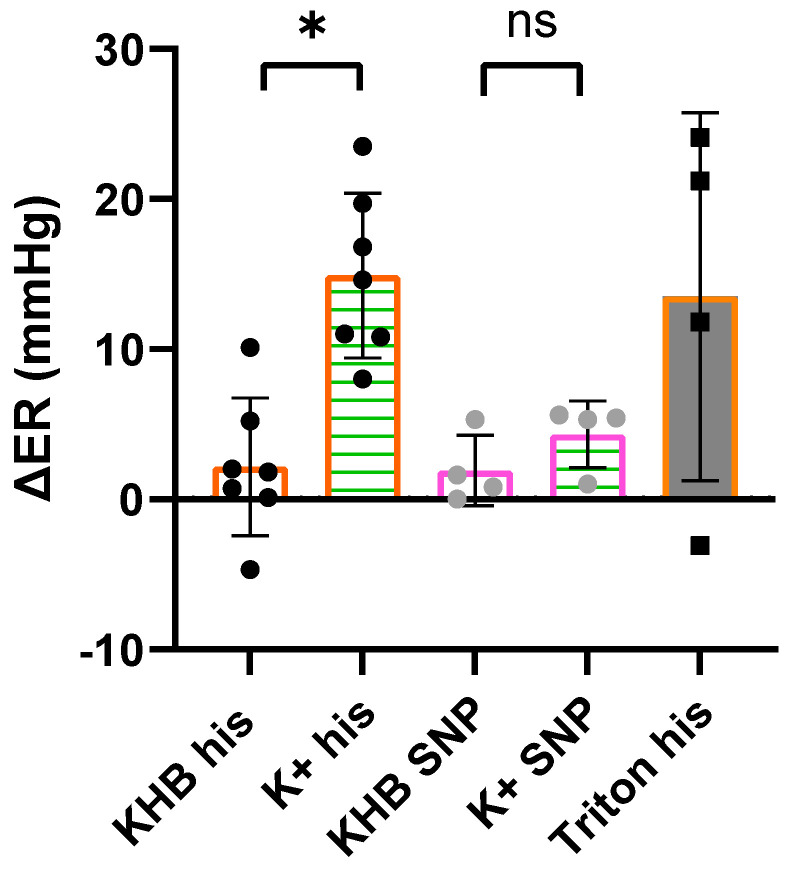
Difference of endothelial response (ΔER = ER_1_ − ER_2_) before (ER_1_) and after (ER_2_) constant flow perfusion (10 min) with Krebs–Henseleit buffer (KHB) alone (white fill) or KHB containing 60 mM KCl (K+, striped green) to 800 nmol histamine (his, orange borders) or 1 µM sodium nitroprusside (SNP, pink borders). As a positive control for ED induction, a bolus of 1 s with 1% triton (grey fill) was used. Data are mean ± SD, *n* = 4 (SNP, triton), *n* = 7 (histamine). One-way ANOVA followed by Šidák’s multiple comparison test. *: *p* < 0.05, ns: not significant (*p* > 0.05).

In order to analyze cardioprotective effects under ED, a published protocol for ED induction by He et al. was modified for the needs of a constant pressure Langendorff system [15]. He et al. perfused Krebs–Henseleit buffer (KHB) containing 60 mM KCl (K+) for 10 min to induce ED in a constant flow Langendorff system. During the establishment of this protocol in a constant pressure system, a direct transfer was not possible. At first, it became evident that perfusion of K+ in constant pressure mode was not able to damage endothelial cells effectively; therefore, shear stress seems to be essential to damage the cells. Similar observations have already been described by Cartier et al. [16]. Furthermore, K+ induces a persistent contraction of the heart with barely noticeable coronary flow, which poses challenges to achieving consistent experimental conditions in a constant pressure system, particularly concerning perfusion time. Consequently, a separate circuit for a constant flow perfusion of K+ was integrated to realize sufficient and reproducible shear stress for cell damage. The final version of the protocol was verified for the induction of ED by measuring the endothelial response (ER) to vasodilators before (ER_1_) and after (ER_2_) perfusion with K+ or with KHB for control conditions.To visualize changes in vasodilator-specific ER before and after perfusion with K+ or KHB alone, the difference in the ERs was calculated (ΔER = ER_1_ − ER_2_, Figure 1). In the case of perfusion with regular KHB, the ΔER for the endothelial-specific vasodilator histamine is low, meaning nearly equal ER before and after perfusion with KHB in response to histamine (KHB his). In contrast, the ΔER for histamine treatment in the analysis of K+ perfusion (K+ his) is high, which indicates a reduced ER after perfusion of K+, and shows a significant difference in comparison to perfusion of KHB. The ΔER values of the endothelial unspecific vasodilator sodium–nitroprusside (SNP) are low and do not differ due to type of perfusion. As a positive control for ED, a 1 s bolus of 1% triton (ED triton) was used and a reduced endothelial response to histamine was measured in comparison to the measurements before treatment with triton.

These results suggest that constant flow perfusion with K+ reduces the ability of the endothelium to respond adequately to histamine by vasodilation, which is a clear sign of ED. The endothelial response to SNP is not modulated by constant flow perfusion with K+ in comparison to perfusion with KHB, suggesting that smooth muscle cell-dependent vasodilation is unaffected by K+. Therefore, this protocol induces successful ED without compromising the functionality of smooth muscle cells.

### 2.2. Conditioning with IL

**Figure 2:**
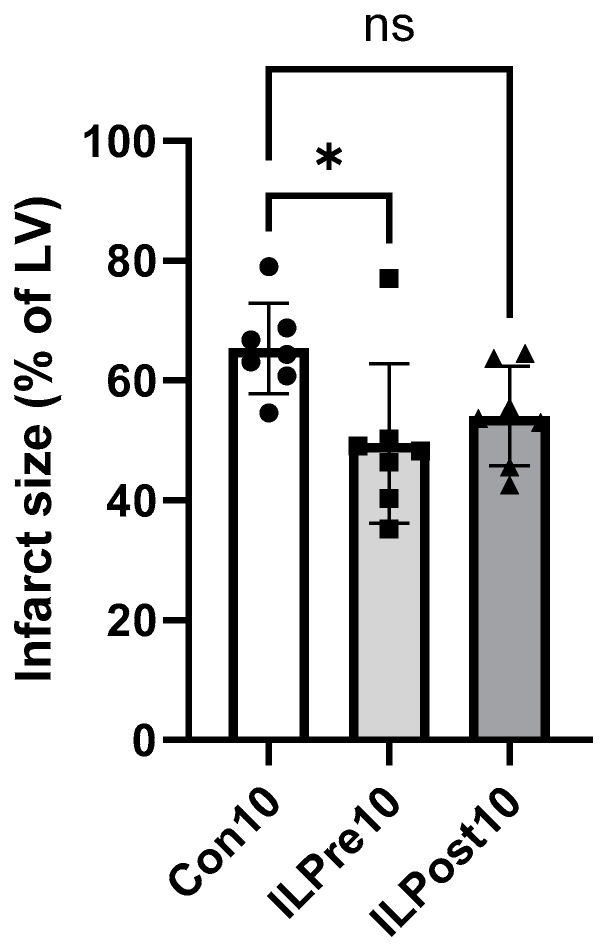
Infarct sizes of hearts after ischemia/reperfusion with pre- or post-treatment for 10 min with 1% Intralipid (IL) or vehicle (Con). LV: left ventricle. Data are mean ± SD, *n* = 7. One-way ANOVA, Dunnett’s multiple comparison test, *: *p* < 0.05, ns: not significant (*p* > 0.05).

To examine the protective effect of treatment with IL against I/R injury, a constant pressure Langendorff system with a separate circuit for perfusion with 1% IL was used. Pre-treatment of 10 min with 1% IL before I/R significantly reduced infarct size compared to an untreated control group (ILPre10: 50 ± 13% vs. Con10: 65 ± 8%, *p* = 0.02, Figure 2). In contrast, infarct size was not significantly reduced by 10 min post-treatment with IL in comparison to the control (ILPost10: 54 ± 8% vs. Con10: 65 ± 8%, *p* = 0.1). Ischemia peaks and body weight and heart weight are not different between the treatment groups and the control group Con10 (Table 1). At baseline measurements, the variables of heart function like heart rate, ventricular diastolic pressure (LVP min), left ventricular systolic pressure (LVP max), left ventricular developed pressure (LVDP), and coronary flow are not different between the control and treatment groups, ensuring equal starting points (Figure S1). After 60 min of reperfusion, a comparison between group Con10 and group ILPre10 reveals a decrease in LVP min (Con10: 79 ± 7 mmHg vs. ILPre10: 66 ± 8 mmHg, *p* = 0.01), which, in combination with an unchanged LVP max, leads to an increase of LVDP (Con10: 21 ± 7 mmHg vs. ILPre10: 37 ± 12 mmHg, *p* = 0.005), while a comparison between Con10 and ILPost10 shows an opposite effect on the two altered variables (LVP min: Con10: 79 ± 7 mmHg vs. ILPost10: 90 ± 8 mmHg, *p* = 0.03; LVDP: Con10: 21 ± 7 mmHg vs. ILPost10: 10 ± 6 mmHg, *p* = 0.048). These results indicate a protective effect on infarct size and LVDP by pre-treatment but not post-treatment with IL, suggesting that IL pre-treatment functions similarly to preconditioning with protective effects against I/R injury.

**Figure 3:**
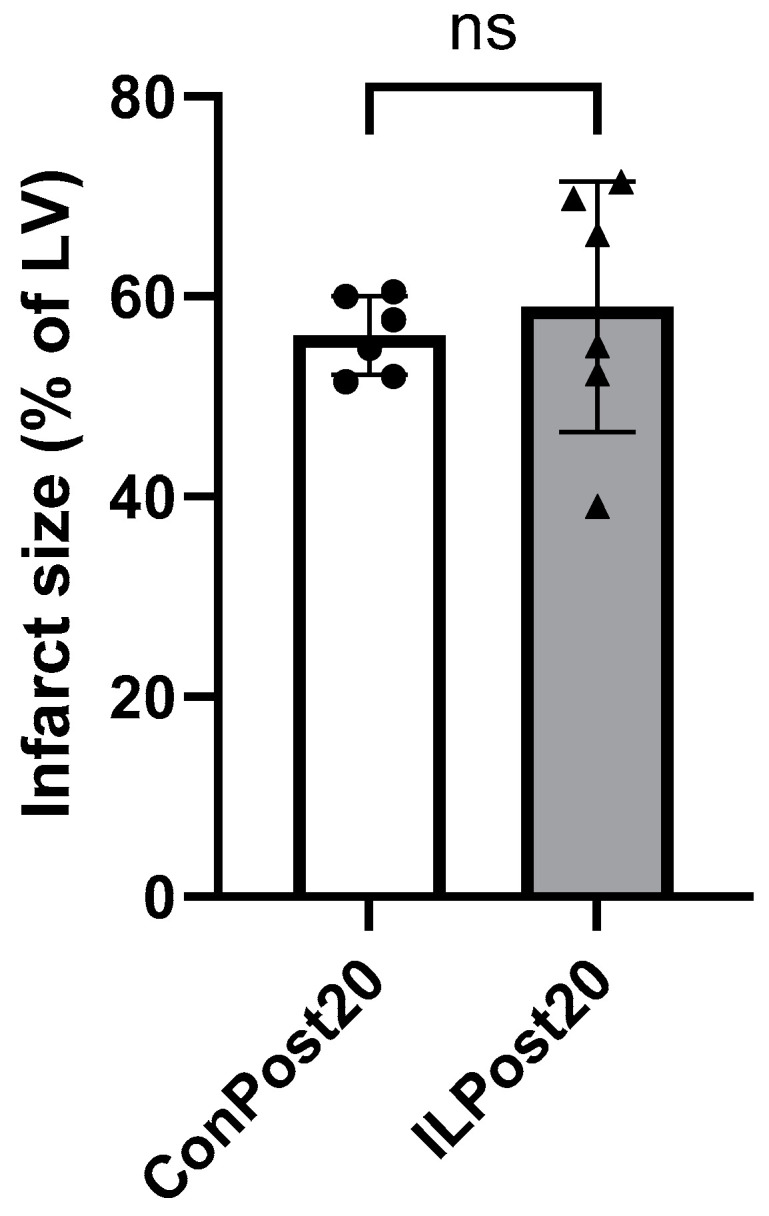
Infarct sizes of hearts after ischemia/reperfusion with post-treatment for 20 min with Intralipid (IL) or vehicle (Con). LV: left ventricle. Data are mean ± SD, *n* = 6. *t*-test; ns: not significant.

Because a slight decrease in infarct size in the ILPost10 group was seen, the period of post-treatment with IL was extended to 20 min in order to enhance a possible protective effect of IL post-treatment. However, no protection could be achieved by prolonging the treatment because infarct sizes of group ILPost20 (60 ± 13%) and the associated control group ConPost20 (56 ± 4%) are comparable in magnitude (Figure 3). Interestingly, wet and dry heart weights are significantly increased in group ILPost20 compared to group ConPost20 despite equal body weights (Table 2). Furthermore, LVP min is significantly increased in group ILPost20 compared to ConPost20 (ILPost20: 93 ± 9 mmHg vs. ConPost20: 79 ± 7 mmHg, *p* = 0.01), resulting in unchanged LVP max in a reduction of LVDP in group ILPost20 (ILPost20: 9 ± 5 mmHg vs. ConPost20: 20 ± 5 mmHg, *p* = 0.003) (Figure S2). Taken together, the extension of post-treatment with IL has no protective effect on infarct size and worsens heart function by reducing LVDP.

### 2.3. Preconditioning with IL under ED

**Figure 4:**
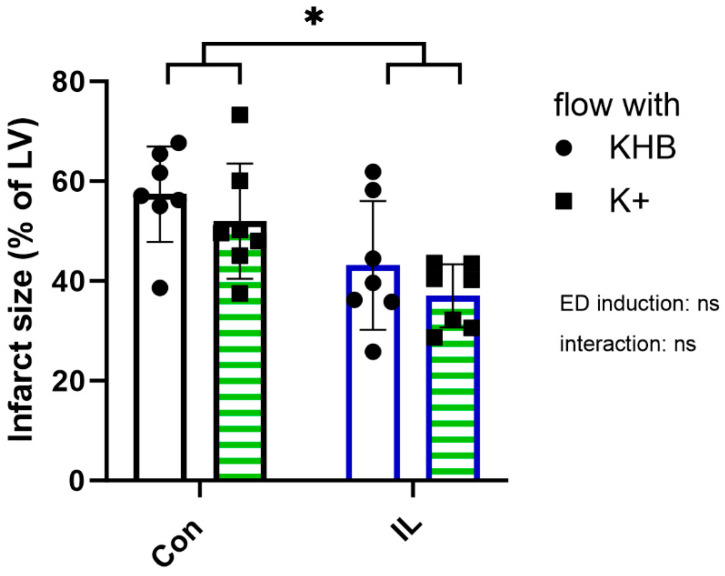
Preconditioning with IL under endothelial dysfunction (ED). Hearts were treated for 10 min before ischemia with 1% Intralipid (IL; blue border) or vehicle (Con; black border). ED was induced by 10 min constant flow perfusion of Krebs–Henseleit buffer (KHB) containing 60 mM KCl (K+; green stripes). The other groups received normal KHB under constant flow conditions (white filling). LV: left ventricle. Data are mean ± SD, *n* = 7. Two-way ANOVA; * *p* < 0.05 for effect by conditioning; ns = not significant for effect by ED induction and interaction (*p* > 0.05).

To verify the cardioprotective effect of preconditioning with IL under ED, the presented model of ED induction was used. As shown in Figure 4, two-way analysis of variance (ANOVA) reveals that constant flow perfusion with K+ has no influence on infarct size in comparison to flow perfusion with KHB alone (ConPre: 57 ± 10%, ConPreK+: 52 ± 12%, ILPre: 43 ± 13%, ILPreK+: 41 ± 12%, *p* = 0.15), while pretreatment with IL significantly reduces infarct size in comparison to control pretreatment with KHB (Con) (*p* = 0.001), and this effect was independent of the induction of ED (*p* = 0.93). Furthermore, ischemia peaks are reduced by preconditioning with IL and by perfusion with K+ (Table 3). Additionally, IL treatment increases dry heart weight. Moreover, LVDP is increased due to a reduced LVP min with a constant LVP max by preconditioning with IL (Figure S3). In addition, endothelial dysfunction increases coronary flow (CF) (Figure S3).

## 3. Discussion

The current research challenge in the field of cardioprotection is to verify and optimize cardioprotective strategies under pathological and, therefore, among more realistic conditions in order to enable a successful transfer from bench to bedside. Here, we present for the first time a refined protocol to induce ED, which can be easily introduced into a conventional constant pressure Langendorff system for studying cardioprotection against I/R injury. The constant pressure Langendorff system is the recommended choice for the quantification of cardiomyocyte death by infarct size, which is the gold standard for measuring cardiomyocyte viability and is considered the preferred primary endpoint in studies focused on cardioprotection [18].

Successful induction of ED by constant flow perfusion of a solution containing high amounts of potassium (K+) is shown here by the reduced endothelial response to the endothelium-dependent vasodilator histamine in comparison to control conditions. Under physiological conditions, histamine mediates vasodilation in endothelial cells via histamine H1 receptors, leading to elevated Ca^2+^ levels and triggering NO production [19]. NO, in turn, diffuses to adjacent smooth muscle cells, eliciting vasodilation [19]. Therefore, both endothelial and smooth muscle cells are required for an adequate endothelial response. A reduced endothelial response to histamine after perfusion with K+ due to damaged smooth muscle cells is unlikely because these cells maintain their functionality under constant flow perfusion with K+, as indicated by unaltered vasodilation in response to the endothelium-independent vasodilator SNP. SNP acts as a prodrug by releasing NO after contact with sulfhydryl groups. In smooth muscle cells, NO binds to NO-sensitive guanylyl cyclases (NO-GC) and stimulates the production of cyclic guanosine monophosphate (cGMP), which, in turn, activates protein kinase G (PKG), finally resulting in the relaxation of vascular smooth muscle cells [20]. Taken together, our protocol successfully induces ED by restricting the vasodilatory functionality of endothelial cells, while preserving the functionality of vascular smooth muscle cells.

An essential consideration for studies investigating cardioprotection against I/R injury is the potential influence of the presented protocol on infarct size, which serves as the gold standard for measuring cardiomyocyte viability [18]. As shown in Figure 4, flow constant perfusion with high potassium amounts has no influence on infarct size. Although a reduction in ischemia peak (Table 3), a sign of a weaker ischemic injury, by K+ perfusion was measured, this does not seem to impact infarct size or heart function. Furthermore, infarct sizes under control conditions are in the order of 50–60% of the total LV area, similar to previous studies without induction of ED, confirming that the established protocol has no impact on infarct size [21,22]. Consequently, this protocol appears suitable for such studies.

To analyze the influence of ED on a known cardioprotective agent, IL was chosen. Prior to this, the cardioprotective effect of IL was verified under untreated conditions in a constant pressure Langendorff system. To generate a 1% IL perfusion solution, only a commercially available concentration of 20% IL was accessible. This low concentration of the stock solution did not allow for the direct addition of IL via a syringe driver into the Langendorff system to perfuse the heart with 1% IL perfusate. However, a detrimental effect could result from the strong dilution of the KHB by 20% IL solution, as the osmotic properties of KHB could be modulated. But this is contradicted by the fact that a concentration of 1% IL in the plasma of patients during treatment against the toxicity of local anesthetics by infusion with 20% IL is recommended and shows hardly any side effects [23]. Therefore, effects on the heart due to dilution of KHB cannot be ruled out but appear to be tolerable for the heart. For perfusion with 1% IL, a separate circuit transporting exclusively this 1% IL perfusate was integrated into the Langendorff system. This approach offered the additional advantage that a perfusion time of 10 min could be reproducibly maintained. Without this setup, significant amounts of IL would have remained in the system due to the viscous properties of IL and its tendency to adhere to tube walls, making a complete IL washout unfeasible. Using this setup, preconditioning with 1% IL for 10 min was cardioprotective against I/R injury by reducing infarct size and improving heart function with elevated LVDP. In contrast, postconditioning with IL induced only a slight reduction of infarct size, while extension to 20 min completely negated a possible protective effect. To our knowledge, this is the first time that a cardioprotective effect of preconditioning with IL in untreated rat hearts has been shown in an in vitro Langendorff system. Ma et al. have already demonstrated the cardioprotective effect of preconditioning with 1% IL by reducing infarct size previously, but the authors were interested in cardioprotection under transverse aortic constriction induced ventricular hypertrophy; therefore, these rats received a sham operation before perfusion [24]. In contrast to preconditioning with IL, a protective effect for postconditioning with 1% IL under comparable conditions to the here-presented study, meaning hearts from untreated rats, was shown earlier. Rahman et al. showed cardioprotective effects by postconditioning with IL in in vitro (mouse) and in vivo (rat) models by infarct size reduction and improved heart function [4]. Interestingly, the authors demonstrated a stronger protective effect in isolated mouse hearts the longer they were treated with IL. This is in clear contrast to the data presented here, as neither 10 min post-treatment with 1% IL nor the extension to 20 min post-treatment protected the heart from I/R injury. One possible explanation could be the variation in the Langendorff system setup because IL was perfused via a separate circuit in our Langendorff system. Notably, other authors did not specify a particular setup for conditioning with IL. In addition, conditioning with IL in the experimental set-up presented here led to a slight increase in the dry weight of the hearts and a paler appearance of the hearts compared to control hearts (Table 1). This observation may imply the deposition of IL within the myocardium. Furthermore, postconditioning with IL for 20 min also resulted in a rise in the wet weight of the hearts (Table 2), indicating the potential onset of edema. The deposition of IL could lead to insufficient coronary flow (CF) during the early reperfusion phase, which, in turn, results in the ineffective clearing of IL from the myocardium and inhibits adequate perfusion later on. The compromised perfusion may account for the observed absence of cardioprotection. Although measurements of CF did not reveal significant differences between the control group and postconditioning groups, a trend towards reduced CF throughout the entire reperfusion phase was discernible (Figures S1 and S2).

The cardioprotective effect of preconditioning with IL remained unaffected by the induction of ED, indicating that protection is mediated independently of an intact endothelium. Similarly, other cardioprotective substances are able to sustain their protective effects after induction of ED. He et al. used a constant flow Langendorff system with perfusion rates of 8–9 mL/min and showed that pretreatment with the α_2_-adrenergic receptor agonist dexmedetomidine (Dex) reduced infarct sizes of hearts after I/R injury compared to control hearts, whereas this protection was independent of ED induction by perfusion with K+ [15]. The authors concluded that the cardioprotective effect of Dex is mediated through a NO-independent mechanism. However, a study using the unspecific eNOS inhibitor L-NAME and the NO-scavenger PTIO (2phenyl-4,4,5,5-tetramethylimidazoline-1-oxyl 3-oxide) on isolated perfused rat hearts revealed a loss of cardioprotection by Dex indicating a NO-dependent protection [25]. Similar contradictory results have also been observed for cardioprotection by oxytocin showing sustained protection under triton-induced ED [26] and a loss of protection by eNOS inhibition [27]. Interestingly, treatment with 1% IL is able to induce NO release in endothelial cells, which, in turn, can be inhibited by L-NAME [28]. Whether these observations hold true in isolated, perfused hearts remains to be elucidated. Nevertheless, in combination with our finding of sustained cardioprotection by IL under ED, the properties of IL fit very well with the properties of the other two substances. However, these findings suggest that an intact endothelium may not be necessary to mediate NO- or NOS-dependent cardioprotection. Cardiomyocytes could serve as a potential source of NO to compensate for the absence of NO from the endothelium, as these cells also express eNOS and neuronal NOS (nNOS) and are therefore able to produce NO required for cardioprotection [29].

One notable limitation of the model presented here is that the protocol induces endothelial dysfunction (ED) within a short time frame of 10 min, whereas ED in patients typically develops over months or years and involves numerous adaptive processes. Therefore, the model used in this study does not account for these gradual adaptations, potentially limiting its relevance to the clinical setting.

Caution should be exercised when transferring findings from the in vitro model of the isolated, perfused heart to clinical situations because systemic influences, such as humoral factors or neuronal interactions, are excluded in the Langendorff system. Consequently, only the response of the isolated heart is measured, which is the major advantage of this model. However, this is also its disadvantage, as interactions that occur within the intact organism are lost and the results may not fully reflect the complexities of in vivo conditions. In the case of ED, disturbed regulation of vascular tone leads to pathological blood supply to organs or tissues, which, in turn, will modulate cardiac function. Therefore, further investigation using in vivo models with ED is mandatory to validate the results from in vitro studies.

## 4. Materials and Methods

### 4.1. Animals

All experiments were performed in accordance with the Guide for the Care and Use of Laboratory Animals, published by the U.S. National Institute of Health (NIH publication No. 85-23, revised 1996) after approval by the local Animal Care and Use Committee of the Heinrich-Heine University, Düsseldorf (project number O27/12). Animals were kept in standardized conditions (food and water provided ad libitum, 12 h day/night cycle, 22 ± 2 °C) and were under the care of the Central Institution for Animal Research and Scientific Animal Welfare (ZETT) of the Heinrich-Heine-University Düsseldorf.

### 4.2. Langendorff System

**Figure 5:**
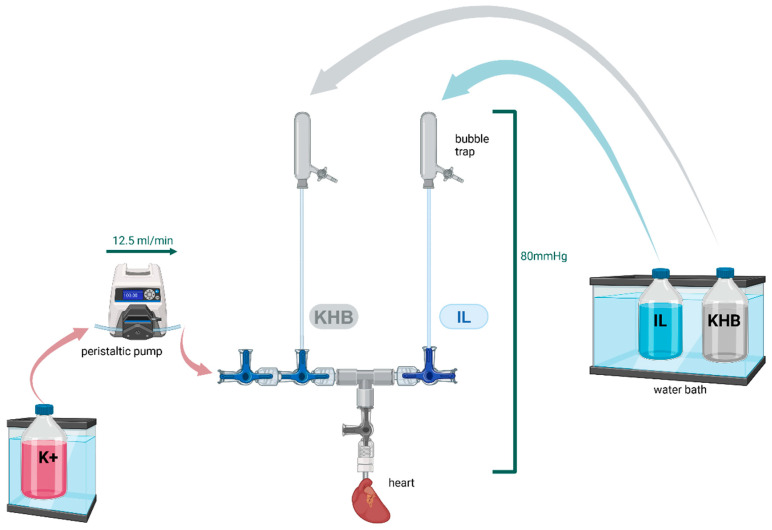
Schematic drawing of the utilized Langendorff system. Krebs–Henseleit buffer (KHB) and KHB containing 1% Intralipid (IL) are perfused in a constant pressure mode (80 mmHg) in separate circuits. To induce endothelial dysfunction, KHB containing 60 mM KCl (K+) is perfused in a constant flow mode. To ensure equivalent control conditions for induction of ED, KHB is perfused in a constant flow mode. The individual modules were switched on or off as required by the respective questions during the experimental setups. Created with BioRender.com.

Male Wistar rats at the age of 2–3 months were randomized into the respective groups and anesthetized with an intraperitoneal injection of 80 mg/kg body weight sodium–pentobarbital (Narcoren, Boehringer Ingelheim, Ingelheim am Rhein, Germany). To avoid agglutination, 3330 IU/kg heparin sodium (Braun SE, Melsungen, Germany) was additionally injected. After decapitation and thoracotomy, the hearts were isolated and mounted on a Langendorff system (Figure 5) and perfused with a modified Krebs–Henseleit solution (KHB; 118 mM NaCl, 4.7 mM KCl, 1.2 mM MgSO_4_, 1.2 mM KH_2_PO_4_, 24.9 mM NaHCO_3_, 2.5 mM CaCl_2_, and 11 mM glucose at 37 °C). In constant pressure mode, a perfusion pressure of 80 mmHg was used. In constant flow mode, a flow of 12.5 mL/min was used. Global ischemia was induced by stopping perfusion with KHB and then immersing the heart into KHB aerated with nitrogen. For reperfusion, heart perfusion with KHB was restored, and the ischemic buffer bath around the heart was removed. Pressure measurements in the left ventricle (LV) were performed via a water-filled balloon placed in the LV. The left ventricular diastolic pressure (LVP min) was set at 4–6 mmHg when using constant pressure mode. Left ventricular pressure was measured continuously, digitized using an analog to digital converter (PowerLab/8SP, ADInstruments Pty Ltd., Castle Hill, Australia) at a sampling rate of 500 Hz, and recorded on a Personal Computer using Labchart 8.0 for Windows (ADInstruments Pty Ltd., Castle Hill, Australia). The following hemodynamic variables were determined: heart rate, LVP min, left ventricular systolic pressure (LVP max), left ventricular developed pressure (LVDP = LVP max − LVP min), and ischemia peak. Coronary flow was measured via the collection of coronary effluent over a set time of 1 min. Under constant flow conditions, coronary perfusion pressure (CPP) was measured via a pressure transducer connected in close proximity to the heart. At the end of the experiment, the weight of the wet hearts was determined and the hearts were frozen for further processing to measure the infarct size.

### 4.3. Experimental Protocol: Verification of ED Induction

**Figure 6:**
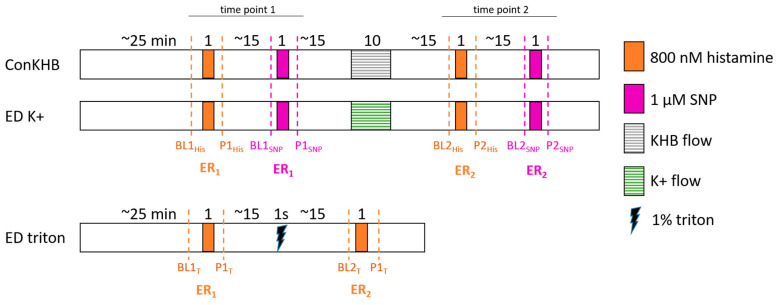
Experimental timeline for characterization of endothelial dysfunction. Dashed blue lines mark measuring points for coronary perfusion pressure (CPP). BL1/2: baseline at time point 1/2, P: after perfusion of vasodilator, His: histamine, T: triton, SNP: sodium–nitroprusside, KHB flow: constant flow perfusion with Krebs–Henseleit buffer, K+ flow: constant flow perfusion with KHB containing 60 mM KCl.

To verify the induction of ED, a constant flow Langendorff system was used. ED was induced by constant flow perfusion (12.5 mL/min) with KHB containing 60 mM KCl (K+). To maintain an equal osmotic condition in the K+ buffer, the amount of NaCl was reduced from 118 mM to 63 mM. Hearts in the control group were perfused under similar conditions but with regular KHB. The experimental design is shown in Figure 6. After equilibration, all hearts were treated with vasodilators before (time point 1) and after (time point 2) perfusion with K+ (ED K+) or with KHB as a control (ConKHB). CPP was measured with a pressure transducer before (BL, baseline) and after (P, post) treatment for 1 min with 800 nmol histamine (#H7125, Merck KGaA, Darmstadt, Germany) or 1 µM sodium–nitroprusside (SNP, #71778, Merck KGaA, Darmstadt, Germany) to induce vasodilation. The concentrations of vasodilators are those used in previous works of other authors [15,30] and were verified by our own dose-response experiments. For each time point and vasodilator, the endothelial response (ER) was determined as ER = BL − P. The difference in endothelial response (ΔER) between both time points (before and after perfusion with K+ or KHB for control conditions) was calculated as ΔER = ER_1_ − ER_2_. As a positive control for a complete destruction of the endothelium, a 1 s bolus of 1% triton (Triton X-100, #T9284, Merck KGaA, Darmstadt, Germany) was used (ED triton).

### 4.4. Experimental Protocol: Experiments with Intralipid

**Figure 7:**
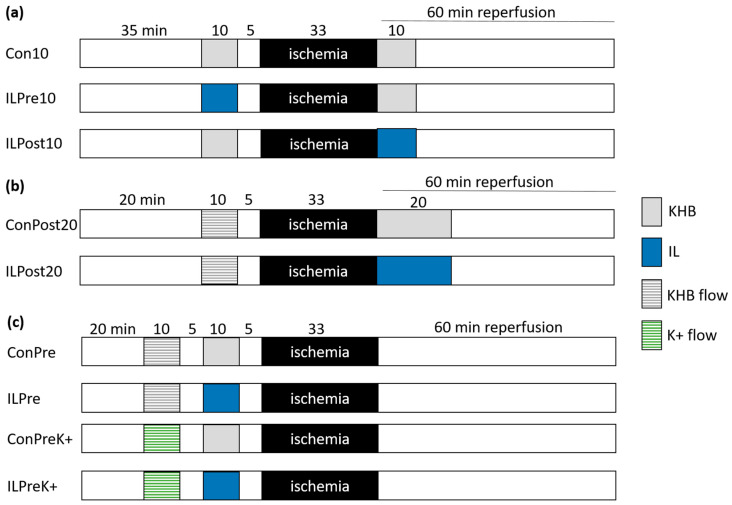
Experimental protocol: conditioning with Intralipid (IL) using a constant pressure Langendorff system with the ability to switch into a constant flow mode for induction of endothelial dysfunction. (**a**) Pre- or postconditioning with IL for 10 min. (**b**) Postconditioning with IL for 20 min. (**c**) Preconditioning with IL under endothelial dysfunction induced by constant flow perfusion of Krebs–Henseleit buffer (KHB) containing 60 mM KCl (K+ flow) and under control condition with constant flow perfusion of KHB alone (KHB flow).

Experiments evaluating the effects of IL treatment on I/R injury via determination of infarct size were performed with a constant pressure Langendorff system (Figure 7). Conditioning with IL was carried out with KHB containing 1% IL (20% Intralipid, Fresenius Kabi, Graz, Austria). IL consists of 20% soybean oil, 1.2% egg yolk phospholipids, and 2.25% glycerine and water. The concentration of 1% IL has been previously demonstrated to exert cardioprotective effects [5,31]. To achieve a consistent duration of IL perfusion and to prevent contamination of the entire Langendorff system with IL, the IL solution was administered through a separate circuit connected in close proximity to the heart. For respective control conditions, normal KHB was perfused via this separate circuit, ensuring that the circuit was without prior contact with IL. For preconditioning, 1% IL was perfused for 10 min before 33 min of global ischemia and 60 min of reperfusion. Before global ischemia, a 5 min washout phase was implemented. For postconditioning, 1% IL was administered for 10 or 20 min at the start of reperfusion.

Firstly, both preconditioning and postconditioning with 1% IL for 10 min were analyzed (Figure 7a). Secondly, the impact of prolonged IL treatment was assessed by postconditioning with 1% IL for 20 min (Figure 7b). The hearts were also perfused with KHB in constant flow mode to ensure comparability for possible subsequent experiments under ED. Thirdly, preconditioning with IL under ED was examined (Figure 7c). ED was induced by constant flow perfusion with K+ via a separate circuit connected in close proximity to the heart using the entire Langendorff system depicted in Figure 5.

### 4.5. Infarct Size Determination

To determine infarct size, frozen hearts were cut into 1 mm slices and stained with 0.75% triphenyltetrazolium chloride (TTC, #37130.02, SERVA Electrophoresis GmbH, Heidelberg, Germany). Afterwards, an investigator blinded to group assignment measured infarcted areas and the total area of the LV using planimetry followed by the determination of infarct size as the percentage of LV area (SigmaScan Pro5, Systat Software Inc., San Jose, CA, USA). The slices were then dried, and the dry weight of the hearts was determined.

### 4.6. Statistics

Comparisons for the verification of successful induction of ED were analyzed using a one-way analysis of variance (ANOVA) followed by Šidák’s multiple comparison test.

A priori sample size analysis with infarct size chosen as the primary endpoint was performed with G*Power 3.1.9.7 [32] and additional statistical analyses were calculated using Prism 10 (GraphPad, Boston, MA, USA). Comparisons for the detection of cardioprotective effects by pre- and postconditioning with IL were conducted using a one-way ANOVA followed by Dunnett’s multiple comparison test for a total sample size of 21 (three groups, effect size 0.75, power 80%, α 0.05). The effectiveness of 20 min of postconditioning with IL was analyzed using the *t*-test; the calculated sample size for detecting a mean difference of 30% with a standard deviation (SD) of 18% in infarct size was six per group (power 80%, α 0.05). Tests to analyze the impact of ED on preconditioning with IL were conducted using a two-way ANOVA. The calculated sample size for detecting effects/interaction on infarct size with two independent variables (ED induction (K+/KHB) and preconditioning (IL/Con)) was 29 (four groups, effect size 0.55, numerator df 1, power 80%, α 0.05). For variables measured longitudinally over time, baseline measurements were analyzed to ensure equivalent starting points. Furthermore, measurements taken at 60 min of reperfusion were analyzed for a comprehensive assessment.

The level of significance was defined as *p* < 0.05; otherwise, the difference was declared as not significant (ns). Data are shown as mean ± SD.

## 5. Conclusions

In conclusion, the method of ED induction presented here offers a valuable approach to studying cardioprotection in a model with an endothelium with impaired function. This facilitates the exploration of the question of whether cardioprotective strategies require a functional endothelium for their efficacy. Notably, preconditioning with IL demonstrates cardioprotective effects independent of endothelial functionality, indicating alternative mechanisms of action.

## Figures and Tables

**Table 1:** Ischemia peaks and body weight and heart weight of experiments with pre- or post-treatment for 10 min with 1% Intralipid (IL) or vehicle (Con). Data are mean ± SD, *n* = 7. One-way ANOVA, Dunnett’s multiple comparison test against Con10, ns: not significant (*p* > 0.05).

	Con10	ILPre10	ILPost10	Significance
Body weight (g)	279 ± 9	294 ± 14	291 ± 15	ns
Ischemia peaks (mmHg)	67 ± 7	56 ± 9	74 ± 10	ns
Heart weight wet (g)	0.93 ± 0.07	0.96 ± 0.06	1.00 ± 0.08	ns
Heart weight dry (mg)	166 ± 24	174 ± 17	174 ± 21	ns

**Table 2:** Ischemia peaks and body and heart weight of experiments with post-treatment for 20 min with Intralipid (IL) or vehicle (Con). Data are mean ± SD, *n* = 6. *t*-test, *: *p* < 0.05, ns: not significant (*p* > 0.05).

	ConPost20	ILPost20
Body weight (g)	286 ± 18	303 ± 13 ^ns^
Ischemia peaks (mmHg)	63 ± 9	78 ± 11 *
Heart weight wet (g)	1.08 ± 0.03	1.23 ± 0.07 *
Heart weight dry (mg)	168 ± 12	207 ± 14 *

**Table 3:** Ischemia peaks and body weight and heart weight of experiments with preconditioning for 10 min with Intralipid (ILPre) or vehicle (ConPre) under control conditions (flow perfusion with normal Krebs–Henseleit buffer (KHB)) or endothelial dysfunction (ED) induced by 10 min constant flow perfusion with KHB containing 60 mM potassium (K+). Ischemia peaks are reduced significantly by preconditioning with IL and by perfusion with K+. Dry heart weight is increased significantly by IL treatment. Data are mean ± SD, *n* = 7. Two-way ANOVA, * *p* < 0.05 for the respective main factors or interaction, ns: not significant (*p* > 0.05).

	ConPre	ILPre	ConPreK+	ILPreK+	Significance
Body weight (g)	286 ± 14	284 ± 12	278 ± 11	284 ± 20	ns
Ischemia peaks (mmHg)	74 ± 8	57 ± 4	55 ± 8	53 ± 8	* conditioning, ED induction, interaction
Heart weight wet (g)	0.99 ± 0.06	1.00 ± 0.05	1.01 ± 0.06	0.98 ± 0.05	ns
Heart weight dry (mg)	166 ± 10	176 ± 10	166 ± 8	174 ± 11	* conditioning

## Data Availability

Dataset available upon request from the authors.

## Supplementary Materials

The supporting information can be downloaded at: https://www.mdpi.com/article/10.3390/ijms252010975/s1.
