# Supplementary material for: Cardioprotection by Preconditioning with Intralipid Is Sustained in a Model of Endothelial Dysfunction for Isolated-Perfused Hearts
Source: Int J Mol Sci. 2024 Oct 12;25(20):10975. doi: 10.3390/ijms252010975 (PMC11507275; doi:10.3390/ijms252010975)
Supplement: Supplementary file 1 [file ijms-25-10975-s001.zip › ijms-3197328-supplementary.pdf]

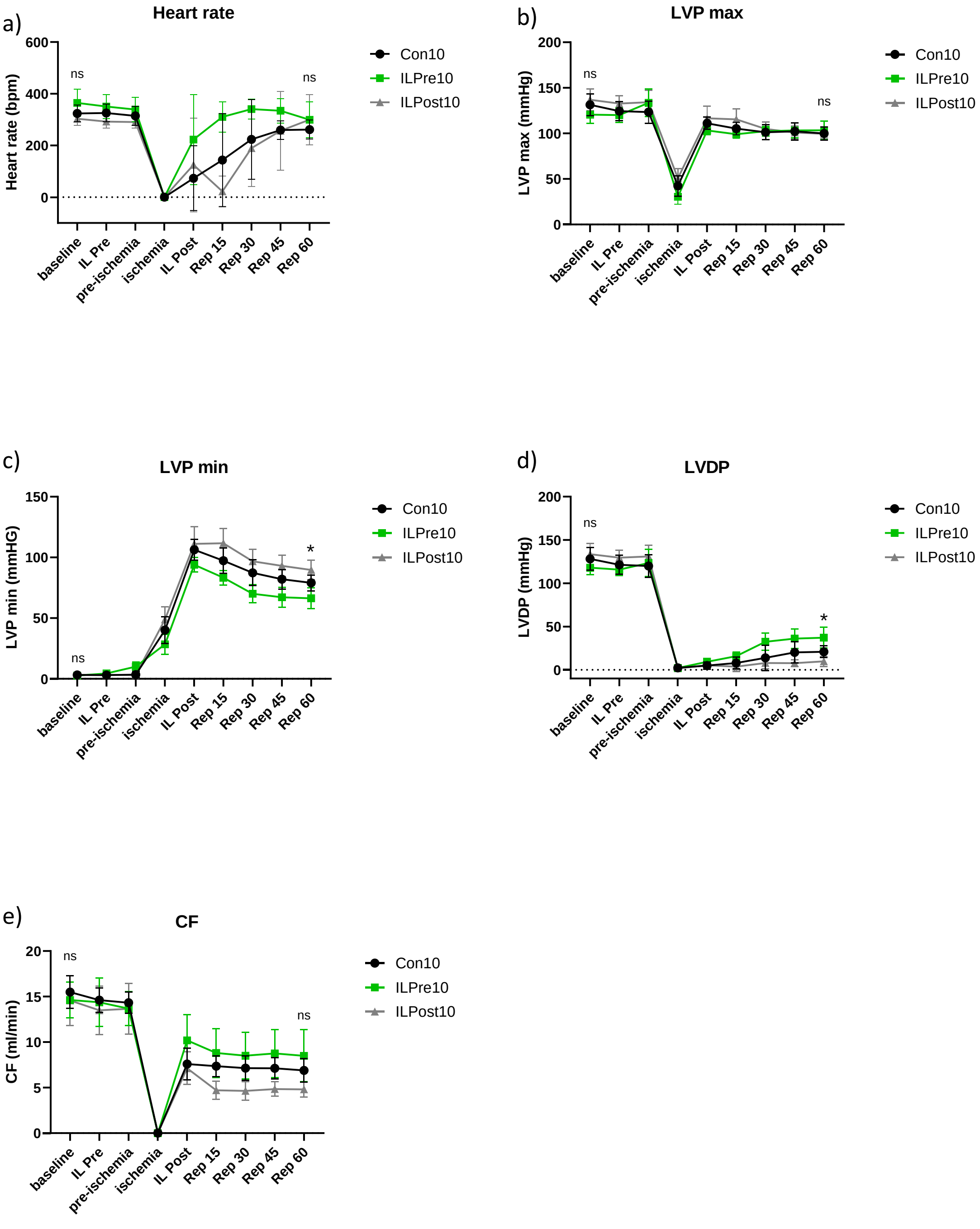

Figure S1 Hemodynamic variables for pre- and posttreatment with Intralipid (IL) or vehicle (Con). a) heart rate; b) LVP max (maximal left ventricular pressure); c) LVP min (minimal LVP); d) LVDP(developed LVP); e) CF (coronary flow). Data are mean +/- SD. Statistical tests were only performed for baseline and at 60 min of reperfusion (Rep 60). One-way ANOVA with Dunnett`s multiple comparison test. * : $p < 0.05$ Con10 vs. IL Pre and Con10 vs. IL Post, ns: not significant for all comparisons ($p > 0.05$). n = 7.

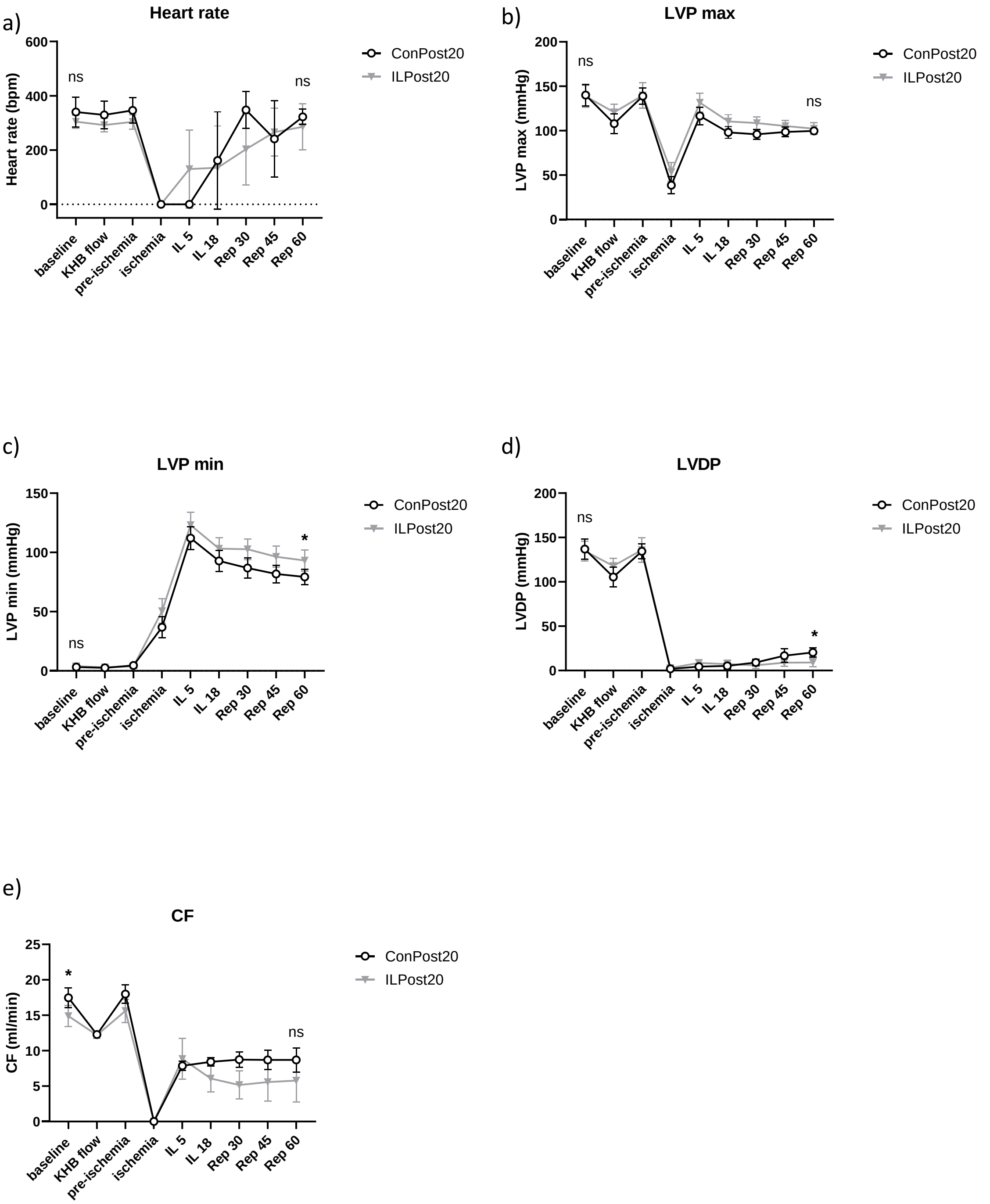

Figure S2 Hemodynamic variables for posttreatment with Intralipid (IL) or vehicle (Con) for 10 minutes. a) heart rate; b) LVP max (maximal left ventricular pressure); c) LVP min (minimal LVP); d) LVDP (developed LVP); CF (coronary flow). Data are mean +/- SD. Statistical tests were only performed for baseline and at 60 min of reperfusion (Rep 60). t-test, * : $p < 0.05$, ns: not significant ($p > 0.05$). n = 6.

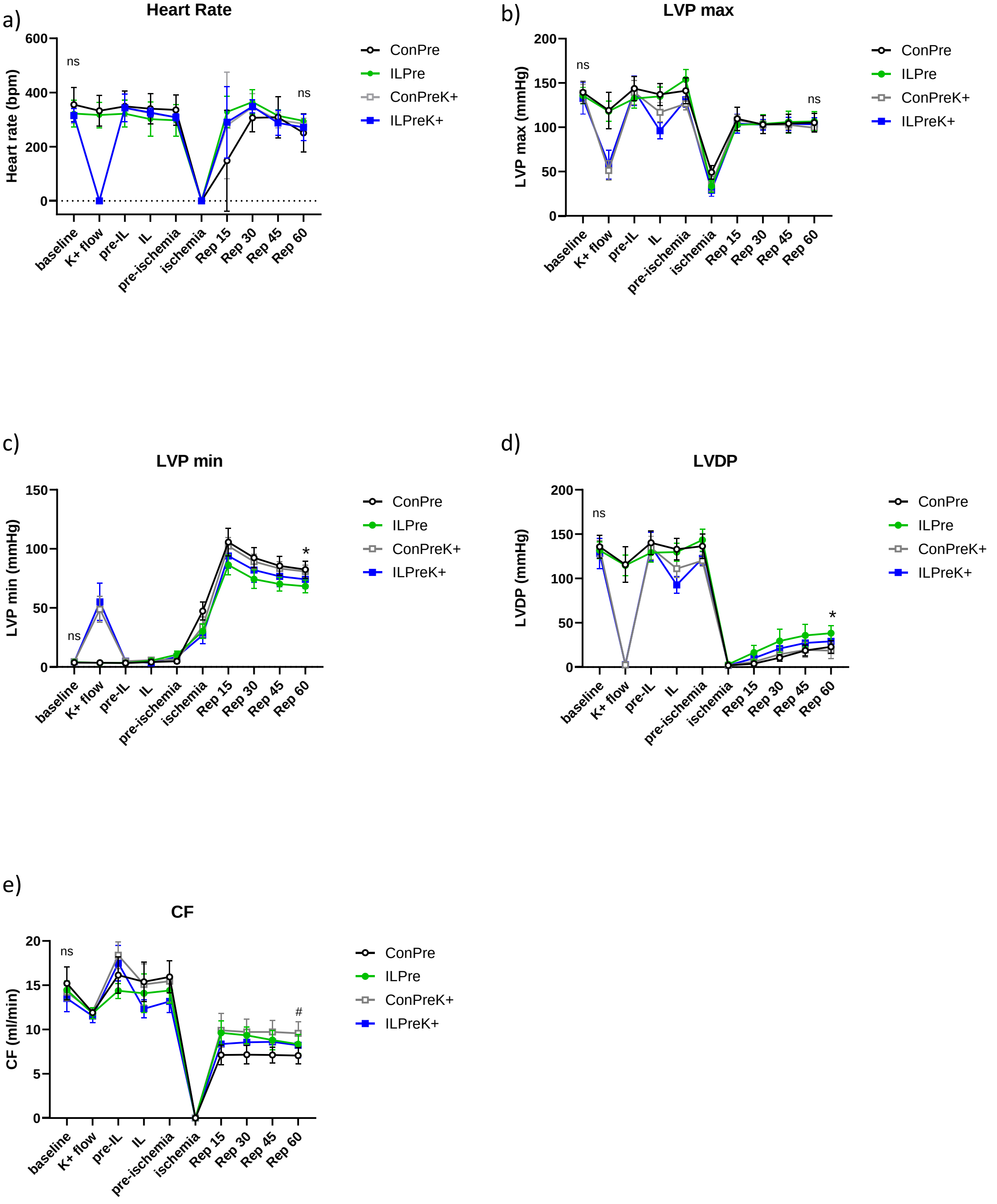

Figure S3 Hemodynamic variables for pretreatment with Intralipid (IL) or vehicle (Con) under endothelial dysfunction (ED) induced by perfusion with KHB containing 60 mM KCl (K+). a) heart rate; b) LVP max (maximal left ventricular pressure); c) LVP min (minimal LVP); d)LVDP (developed LVP); e) CF (coronary flow). Data are mean ± SD. Statistical tests were only performed for baseline and at 60 min of reperfusion (Rep 60). Two-way ANOVA, * : $p < 0.05$ for effect by conditioning (ConPre vs ILPre), # : $p < 0.05$ for effect by ED induction and interaction. ns: not significant for all comparisons ($p > 0.05$). n = 7.
